# 5,6-Diamino-1,3-benzodithiole-2-thione

**DOI:** 10.1107/S1600536810046532

**Published:** 2010-11-13

**Authors:** Fang-Ming Wang

**Affiliations:** a School of Materials Science and Engineering, Jiangsu University of Science and Technology, Zhenjiang 212003, People’s Republic of China

## Abstract

The mol­ecule of the title compound, C_7_H_6_N_2_S_3_, is almost planar, the dihedral angle between the benzene plane and the 1,3-dithiole-2-thione plane being 2.21 (6)°. In the crystal, mol­ecules are linked by inter­molecular N—H⋯S and N—H⋯N hydrogen bonds into a three-dimensional network. The crystal packing also exhibits weak inter­molecular S⋯S inter­actions [3.5681 (9) Å].

## Related literature

For background to tetra­thio­fulvalene and its derivatives, see: Yamada & Sugimoto (2004 ▶). For the synthesis and properties of tetra­thio­fulvalene and its derivatives, see: Otsubo & Takimiya (2004 ▶); Krief (1986 ▶); Jia *et al.* (2007 ▶).
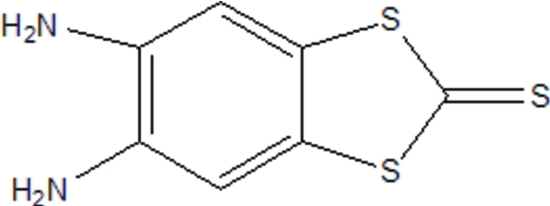


## Experimental

### 

#### Crystal data

C_7_H_6_N_2_S_3_
*M* *_r_* = 214.35
Monoclinic, 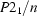

*a* = 5.7695 (9) Å
*b* = 7.6130 (11) Å
*c* = 19.993 (3) Å
β = 94.265 (2)°
*V* = 875.7 (2) Å^3^
*Z* = 4
Mo *K*α radiation
μ = 0.79 mm^−1^
*T* = 291 K
0.35 × 0.10 × 0.05 mm

#### Data collection

Bruker SMART CCD area-detector diffractometer
Absorption correction: multi-scan (*SADABS*; Bruker, 2000 ▶) *T*_min_ = 0.910, *T*_max_ = 0.961
4517 measured reflections
1702 independent reflections
1521 reflections with *I* > 2σ(*I*)
*R*_int_ = 0.029

#### Refinement

*R*[*F* ^2^ > 2σ(*F* ^2^)] = 0.032
*wR*(*F* ^2^) = 0.097
*S* = 1.00
1702 reflections
133 parameters
H atoms treated by a mixture of independent and constrained refinement
Δρ_max_ = 0.21 e Å^−3^
Δρ_min_ = −0.29 e Å^−3^

### 

Data collection: *SMART* (Bruker, 2000 ▶); cell refinement: *SAINT* (Bruker, 2000 ▶); data reduction: *SAINT*; program(s) used to solve structure: *SHELXTL* (Sheldrick, 2008 ▶); program(s) used to refine structure: *SHELXTL*; molecular graphics: *SHELXTL*; software used to prepare material for publication: *SHELXTL*.

## Supplementary Material

Crystal structure: contains datablocks I, global. DOI: 10.1107/S1600536810046532/rz2516sup1.cif

Structure factors: contains datablocks I. DOI: 10.1107/S1600536810046532/rz2516Isup2.hkl

Additional supplementary materials:  crystallographic information; 3D view; checkCIF report

## Figures and Tables

**Table 1:** Hydrogen-bond geometry (Å, °)

*D*—H⋯*A*	*D*—H	H⋯*A*	*D*⋯*A*	*D*—H⋯*A*
N1—H2*A*⋯S3^i^	0.84 (4)	2.87 (4)	3.711 (3)	176 (3)
N2—H3*A*⋯N1^ii^	0.83 (3)	2.45 (3)	3.226 (3)	156 (3)
N2—H4*A*⋯S3^iii^	0.84 (3)	2.90 (3)	3.588 (2)	141 (3)

Symmetry codes: (i) 
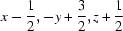
; (ii) 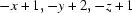; (iii) 
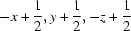
.

**Fig. 1.**
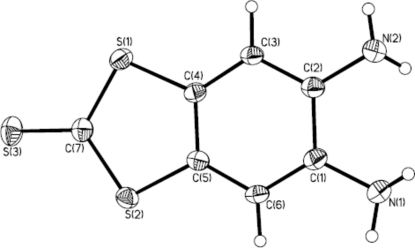
The molecular structure of the title compound with displacement ellipsoids drawn at the 50% probability level.

**Fig. 2.**
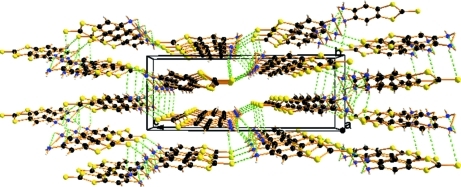
Crystal packing of the title compound viewed along the a axis. Intermolecular hydrogen bonds are shown as dashed lines.

## supplementary crystallographic information

### Comment

Tetrathiofulvalene (TTF) and its derivatives are successfully used as versatile building blocks for charge-transfer salts, giving rise to organic conductors and superconductors because of their unique π-donor properties (Yamada & Sugimoto, 2004). Extensive reviews on the synthesis and properties of TTF and its derivatives have been published (Otsubo & Takimiya, 2004; Krief, 1986). 1,3-Dithiole-2-thiones are a key intermediates in TTF synthesis routes (Jia *et al.*, 2007). The synthesis and crystal structure of the title compound is reported herein.

The molecular structure of the title compound is shown in Fig. 1. The dihedral angle between the benzene plane and the 1,3-dithiole-2-thione plane is 2.21 (6)°. The moleculess are linked by the intermolecular N–H···S and N–H···N hydrogen bonds (Table 1) and S···S weak interactions (3.5681 (9) Å) into a three-dimensional network (Fig. 2).

### Experimental

1,2-Diaminobenzene-4,5-bis(thiocyanate) (10 mmol) was added to a degassed solution of Na_2_S.9H_2_O (33 mmol) in water (100 mL), and the mixture was heated to 70 °C for an hour to produce a clear brownish solution. The mixture was cooled to 50 °C, and CS_2_ (1.4 ml, 23.2 mmol) was slowly added dropwise. The mixture was stirred for two hours at 50 °C and for further three hours at room temperature. The precipitate was filtered off, washed with water, and air-dried. The crude product was purified by flash column chromatography to give the title compound as a yellow powder (yield 50%). Single crystals of the title compound suitable for X-ray analysis were obtained by slow evaporation of an ethyl acetate solution at room temperature for two weeks.

### Refinement

All H atoms were located in a difference Fourier map and refined freely.

### Crystal data

**Table:**

C_7_H_6_N_2_S_3_	*F*(000) = 440
*M**_r_* = 214.35	*D*_x_ = 1.626 Mg m^−^^3^
Monoclinic, *P*2_1_/*n*	Mo *K*α radiation, λ = 0.71073 Å
Hall symbol: -P 2yn	Cell parameters from 2863 reflections
*a* = 5.7695 (9) Å	θ = 3.1–27.3°
*b* = 7.6130 (11) Å	µ = 0.79 mm^−^^1^
*c* = 19.993 (3) Å	*T* = 291 K
β = 94.265 (2)°	Block, yellow
*V* = 875.7 (2) Å^3^	0.35 × 0.10 × 0.05 mm
*Z* = 4	

### Data collection

**Table:**

Bruker SMART CCD area-detector diffractometer	1702 independent reflections
Radiation source: sealed tube	1521 reflections with *I* > 2σ(*I*)
graphite	*R*_int_ = 0.029
phi and ω scans	θ_max_ = 26.0°, θ_min_ = 2.9°
Absorption correction: multi-scan (*SADABS*; Bruker, 2000)	*h* = −6→7
*T*_min_ = 0.910, *T*_max_ = 0.961	*k* = −9→9
4517 measured reflections	*l* = −20→24

### Refinement

**Table:**

Refinement on *F*^2^	Primary atom site location: structure-invariant direct methods
Least-squares matrix: full	Secondary atom site location: difference Fourier map
*R*[*F*^2^ > 2σ(*F*^2^)] = 0.032	Hydrogen site location: inferred from neighbouring sites
*wR*(*F*^2^) = 0.097	H atoms treated by a mixture of independent and constrained refinement
*S* = 1.00	*w* = 1/[σ^2^(*F*_o_^2^) + (0.0558*P*)^2^ + 0.4807*P*] where *P* = (*F*_o_^2^ + 2*F*_c_^2^)/3
1702 reflections	(Δ/σ)_max_ < 0.001
133 parameters	Δρ_max_ = 0.21 e Å^−^^3^
0 restraints	Δρ_min_ = −0.29 e Å^−^^3^

### Special details

**Table:**

Geometry. All e.s.d.'s (except the e.s.d. in the dihedral angle between two l.s. planes) are estimated using the full covariance matrix. The cell e.s.d.'s are taken into account individually in the estimation of e.s.d.'s in distances, angles and torsion angles; correlations between e.s.d.'s in cell parameters are only used when they are defined by crystal symmetry. An approximate (isotropic) treatment of cell e.s.d.'s is used for estimating e.s.d.'s involving l.s. planes.
Refinement. Refinement of *F*^2^ against ALL reflections. The weighted *R*-factor *wR* and goodness of fit *S* are based on *F*^2^, conventional *R*-factors *R* are based on *F*, with *F* set to zero for negative *F*^2^. The threshold expression of *F*^2^ > σ(*F*^2^) is used only for calculating *R*-factors(gt) *etc*. and is not relevant to the choice of reflections for refinement. *R*-factors based on *F*^2^ are statistically about twice as large as those based on *F*, and *R*- factors based on ALL data will be even larger.

### Fractional atomic coordinates and isotropic or equivalent isotropic displacement parameters (Å^2^)

**Table:**

	*x*	*y*	*z*	*U*_iso_*/*U*_eq_	
C1	0.5326 (4)	0.8170 (3)	0.39432 (11)	0.0350 (5)	
C2	0.3149 (4)	0.8999 (3)	0.37772 (11)	0.0334 (4)	
C3	0.2459 (4)	0.9342 (3)	0.31133 (11)	0.0316 (4)	
C4	0.3887 (3)	0.8872 (3)	0.26093 (10)	0.0305 (4)	
C5	0.6025 (4)	0.8080 (3)	0.27734 (10)	0.0312 (4)	
C6	0.6739 (4)	0.7722 (3)	0.34410 (11)	0.0332 (4)	
C7	0.5787 (4)	0.8467 (3)	0.14729 (11)	0.0369 (5)	
H3	0.097 (5)	0.992 (3)	0.3011 (12)	0.038 (6)*	
H6	0.820 (4)	0.719 (3)	0.3564 (12)	0.040 (6)*	
H1A	0.724 (6)	0.720 (4)	0.4636 (16)	0.073 (10)*	
H2A	0.490 (7)	0.760 (4)	0.4841 (17)	0.066 (10)*	
H3A	0.250 (5)	0.978 (4)	0.4623 (15)	0.058 (9)*	
H4A	0.060 (6)	0.999 (4)	0.4153 (15)	0.057 (8)*	
N1	0.6017 (4)	0.7889 (3)	0.46186 (11)	0.0482 (5)	
N2	0.1723 (4)	0.9361 (3)	0.42920 (11)	0.0465 (5)	
S1	0.32535 (10)	0.93005 (7)	0.17590 (3)	0.03893 (19)	
S2	0.76856 (9)	0.76272 (7)	0.20996 (3)	0.03781 (19)	
S3	0.63040 (13)	0.84730 (10)	0.06737 (3)	0.0540 (2)	

### Atomic displacement parameters (Å^2^)

**Table:**

	*U*^11^	*U*^22^	*U*^33^	*U*^12^	*U*^13^	*U*^23^
C1	0.0329 (11)	0.0347 (10)	0.0364 (11)	0.0004 (8)	−0.0046 (8)	0.0003 (8)
C2	0.0290 (10)	0.0325 (10)	0.0383 (11)	0.0010 (8)	0.0003 (8)	−0.0026 (8)
C3	0.0246 (10)	0.0318 (10)	0.0380 (11)	0.0016 (8)	−0.0013 (8)	−0.0015 (8)
C4	0.0274 (10)	0.0288 (9)	0.0347 (10)	−0.0011 (8)	−0.0011 (8)	0.0014 (8)
C5	0.0274 (10)	0.0276 (9)	0.0385 (11)	−0.0005 (8)	0.0006 (8)	−0.0028 (8)
C6	0.0261 (10)	0.0326 (10)	0.0399 (11)	0.0047 (8)	−0.0037 (8)	0.0011 (8)
C7	0.0378 (12)	0.0344 (11)	0.0382 (12)	−0.0078 (9)	0.0023 (9)	−0.0027 (8)
N1	0.0430 (13)	0.0641 (13)	0.0362 (11)	0.0132 (11)	−0.0044 (9)	0.0018 (10)
N2	0.0384 (11)	0.0626 (14)	0.0382 (11)	0.0120 (10)	0.0008 (9)	−0.0053 (10)
S1	0.0351 (3)	0.0450 (3)	0.0360 (3)	0.0018 (2)	−0.0018 (2)	0.0058 (2)
S2	0.0304 (3)	0.0421 (3)	0.0412 (3)	0.0014 (2)	0.0044 (2)	−0.0036 (2)
S3	0.0592 (4)	0.0666 (4)	0.0368 (4)	−0.0103 (3)	0.0088 (3)	−0.0028 (3)

### Geometric parameters (Å, °)

**Table:**

C1—C6	1.382 (3)	C5—S2	1.745 (2)
C1—N1	1.396 (3)	C6—H6	0.95 (3)
C1—C2	1.423 (3)	C7—S3	1.647 (2)
C2—C3	1.382 (3)	C7—S2	1.725 (2)
C2—N2	1.392 (3)	C7—S1	1.730 (2)
C3—C4	1.395 (3)	N1—H1A	0.88 (4)
C3—H3	0.97 (3)	N1—H2A	0.84 (4)
C4—C5	1.390 (3)	N2—H3A	0.83 (3)
C4—S1	1.743 (2)	N2—H4A	0.84 (3)
C5—C6	1.394 (3)		
			
C6—C1—N1	121.6 (2)	C1—C6—C5	119.9 (2)
C6—C1—C2	119.9 (2)	C1—C6—H6	118.4 (14)
N1—C1—C2	118.5 (2)	C5—C6—H6	121.7 (15)
C3—C2—N2	121.9 (2)	S3—C7—S2	123.70 (14)
C3—C2—C1	119.6 (2)	S3—C7—S1	122.57 (14)
N2—C2—C1	118.4 (2)	S2—C7—S1	113.73 (13)
C2—C3—C4	120.15 (19)	C1—N1—H1A	108 (2)
C2—C3—H3	118.3 (14)	C1—N1—H2A	112 (2)
C4—C3—H3	121.6 (14)	H1A—N1—H2A	118 (3)
C5—C4—C3	120.12 (19)	C2—N2—H3A	110 (2)
C5—C4—S1	115.43 (16)	C2—N2—H4A	111 (2)
C3—C4—S1	124.37 (16)	H3A—N2—H4A	114 (3)
C4—C5—C6	120.3 (2)	C7—S1—C4	97.64 (10)
C4—C5—S2	115.60 (16)	C7—S2—C5	97.59 (10)
C6—C5—S2	124.07 (16)		
			
C6—C1—C2—C3	0.3 (3)	N1—C1—C6—C5	−177.7 (2)
N1—C1—C2—C3	178.0 (2)	C2—C1—C6—C5	−0.2 (3)
C6—C1—C2—N2	177.2 (2)	C4—C5—C6—C1	−0.6 (3)
N1—C1—C2—N2	−5.2 (3)	S2—C5—C6—C1	178.36 (16)
N2—C2—C3—C4	−176.5 (2)	S3—C7—S1—C4	180.00 (14)
C1—C2—C3—C4	0.2 (3)	S2—C7—S1—C4	0.34 (13)
C2—C3—C4—C5	−1.0 (3)	C5—C4—S1—C7	0.28 (17)
C2—C3—C4—S1	−177.76 (16)	C3—C4—S1—C7	177.22 (18)
C3—C4—C5—C6	1.1 (3)	S3—C7—S2—C5	179.66 (14)
S1—C4—C5—C6	178.20 (16)	S1—C7—S2—C5	−0.69 (13)
C3—C4—C5—S2	−177.88 (15)	C4—C5—S2—C7	0.90 (17)
S1—C4—C5—S2	−0.8 (2)	C6—C5—S2—C7	−178.06 (18)

### Hydrogen-bond geometry (Å, °)

**Table:**

*D*—H···*A*	*D*—H	H···*A*	*D*···*A*	*D*—H···*A*
N1—H2A···S3^i^	0.84 (4)	2.87 (4)	3.711 (3)	176 (3)
N2—H3A···N1^ii^	0.83 (3)	2.45 (3)	3.226 (3)	156 (3)
N2—H4A···S3^iii^	0.84 (3)	2.90 (3)	3.588 (2)	141 (3)

Symmetry codes: (i) *x*−1/2, −*y*+3/2, *z*+1/2; (ii) −*x*+1, −*y*+2, −*z*+1; (iii) −*x*+1/2, *y*+1/2, −*z*+1/2.
